# Efficient *β*-Carboline Alkaloid-Based Probe for Highly Sensitive Imaging of Endogenous Glutathione in Wheat Germ Tissues

**DOI:** 10.1155/2020/8675784

**Published:** 2020-09-15

**Authors:** Xiaohui Ji, Dan Zhang, Le Li, Lingxia Jin, Rui Wu

**Affiliations:** ^1^Shaanxi Province Key Laboratory of Catalytic Foundation and Application, School of Chemistry and Environment Science, Shaanxi University of Technology, Hanzhong 723001, China; ^2^Shaanxi Key Laboratory of Industrial Automation, School of Mechanical Engineering, Shaanxi University of Technology, Hanzhong 723001, China

## Abstract

Discriminative detection of GSH is achieved by employing a highly sensitive and selective fluorescent probe (**KL-DN**) that bears *β*-carboline alkaloid as a potential fluorophore and an azide group as the recognition unit. A rapid fluorescence off-on change is caused by special redox reaction; **KL-DN** has the capability of monitoring endogenous GSH in wheat germ tissues, indicating that this probe holds great potential for biological applications in plant tissues.

## 1. Introduction

Wheat germ containing abundant GSH, a nutrition with high protein, high vitamin E, and low cholesterol, is beneficial to human health [1]. Interestingly, research indicates that GSH directly or indirectly involves in many functional activities in wheat seedling leaves under osmotic stress, including the metabolism of drugs and hormone in the plant cells and the biosynthesis of DNA, RNA, and protein [2–4]. As an essential endogenic antioxidant, GSH can protect plant cells from oxidative damage [5]. Accordingly, the development of economical and effective methods for detection of GSH in wheat germ system is of considerable importance for better understanding of its physiological functions and has become the significant subject of current chemical research.

**Figure 1:**
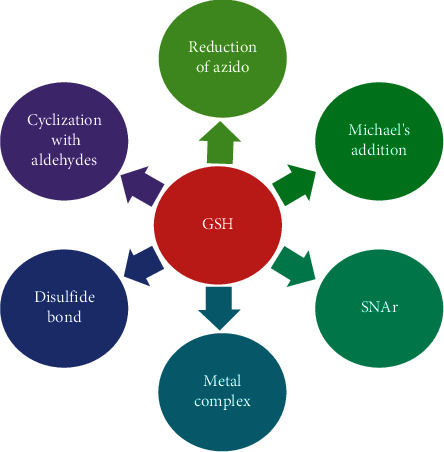
The reaction types of GSH.

Among the multiple analytical methods including electrochemical voltammetry, optical sensing, high-performance liquid chromatography, and mass spectrometry, fluorescence analysis is more popular because of its high safety, low cost, simplicity of operation, and noninvasiveness [6–8]. In consideration of the sensitivity and great bioimaging potential of fluorescence detection [9], considerable effort had been paid in the development of fluorescent probes for GSH detection based on different reactive mechanisms (Figure 1). For instance, Xie et al. introduced that the SH residue of GSH, and the aldehyde group of the fluorescent probe underwent the addition reaction resulting in the open-ring form in acid conditions [10]. Disulfide bonds have been abundantly exploited to measure GSH for mediating the extracellular release of drugs [11–14]. The disadvantages of long reaction time and high limit of detection were the ubiquitous challenge. The fluorescent probes with better SNAr leaving groups were susceptible to SNAr substitution by sulfhydryl thiols with poor selectivity [15–20]. Qu and coworkers reported a fluorescent turn-on method of Ag-S-GF taking advantage of specific interactions between sulfur and silver cation [21]. The mechanisms of recognizing GSH also included the selective cleavage of the selenium-nitrogen bond, Michael's addition, the reduction of azido, and others [22–30]. Although these researches greatly accelerated the development of fluorescent detection, the design of a highly sensitive chemosensor on the basis of natural product structure for recognizing GSH in situ in plant organisms is still a significant challenge.

**Scheme 1:**
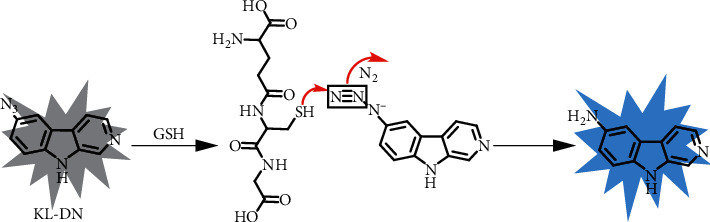
Chemical structure of **KL-DN** and the proposed mechanism.

As an active natural product, *β*-carboline alkaloids with good biocompatibility are composed of indole ring and pyridine ring, showing broad and significant biological activities [31–33]. In addition to these advantages, *β*-carboline alkaloids are capable of emitting strong fluorescence [34]. Unfortunately, very little research on using *β*-carboline alkaloids as fluorescent probes had been reported [35]. Selecting *β*-carboline alkaloid as a potential fluorophore and an azide group as the GSH recognition unit, we reported herein the design, synthesis, and evaluation of the turn-on probe **KL-DN** for rapidly discriminating GSH (Scheme 1). This novel probe offered exceedingly rapid (∼2 min) and low concentration (∼92 nM) (3s/slope) detection of GSH under physiological conditions without the need for excessively high probe/analyte ratios to achieve rapid probe activation. **KL-DN** exhibited remarkable selectivity toward GSH and Cys in comparison to other amino acids and the gasotransmitter H_2_S. Importantly, due to its design, **KL-DN** is taken up and activated in a very rapid fashion, so as to offer high-speed imaging of endogenous GSH in plant tissues and human cells with a high signal-to-background ratio.

## 2. Results and Discussion

### 2.1. Design and Synthesis of **KL-DN**

The **KL-DN** was prepared through the synthetic route outlined in Figure S1. We selected the inexpensive raw material (tryptophan) to synthesize compound **1** by Pictet–Spengler reaction. By means of oxidation reaction and nitrification, the formed compound **3** was changed into compound **4** in the presence of Pd/C and hydrazine hydrate. Then the amine group of compound **4** was further converted to azide group of **KL-DN** with 53% yield. The final product was characterized by ^1^H NMR, ^13^C NMR, and MS spectrum (Figures S5–S7).

### 2.2. Evaluation of Optical Responses to GSH

**Figure 2:**
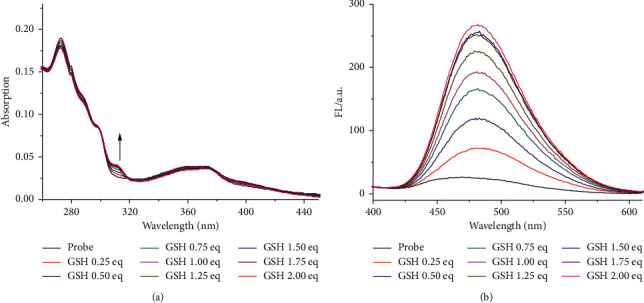
(a) The absorption spectral changes of **KL-DN** (10 *μ*M) upon addition of 2 eq GSH. (b) The fluorescence spectral changes of **KL-DN** (10 *μ*M) upon addition of GSH, *λ*_ex_ = 310 nm. All the solutions are in DMSO/PBS buffer (1:1, v/v, pH 7.4).

The ability of **KL-DN** for sensing GSH was investigated in 10 mM PBS buffer (pH = 7.4) with 50% DMSO (v/v) at 25°C. Under this condition, **KL-DN** (10 *μ*M) showed maximum absorption at 275 nm and displayed weak fluorescence. Upon addition of 2 eq GSH, the absorption intensity at 310 nm increased, which indicated that **KL-DN** was capable of reacting with GSH. According to the absorption spectrum, we choose 310 nm as the excitation wavelength. The fluorescence intensity at a new red-shifted emission peak (487 nm) evidently enhanced as high as 20-fold upon successive addition of GSH with good linear relationship and large stokes shifts (Figure 2(b)) (Figure S8A). The fluorescence quantum yield of **KL-DN** with the addition of GSH increased from 0.0013 to 0.038 with quinine sulfate as reference. The solvent system for measuring the fluorescence quantum yield of the probe **KL-DN** was in DMSO/PBS buffer (1:1). Meanwhile, the solution started to show a bright blue fluorescence, which is highly visible to the naked eye (observed under a portable 365 nm UV light) (Figure S8B). The Job's plot through fluorescence intensity changes indicated a 1:1 reactive ratio of probe with GSH (Figure S9). To investigate the effects of pH on the fluorescent response of **KL-DN** toward GSH, fluorescence intensity changes were measured from pH 2–12. In the presence of GSH, the probe **KL-DN** showed strong fluorescence in the range of 6–8 (Figure S10), which demonstrated that **KL-DN** could be used to detect GSH in real samples.

### 2.3. Selectivity of **KL-DN** to GSH

**Figure 3:**
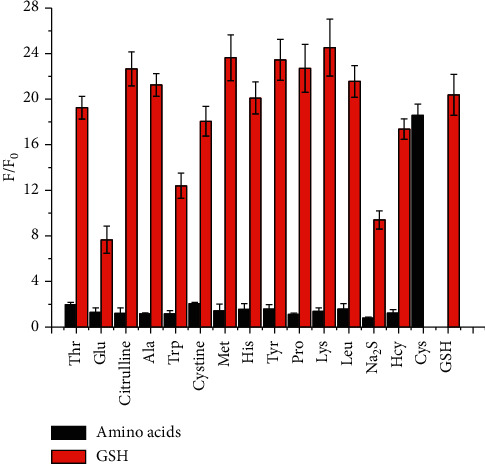
Fluorescence responses of **KL-DN** (10 *μ*M) to different amino acids (20 *μ*M) (blank bar) in DMSO/PBS buffer (1:1, v/v, 10 mM, pH 7.4), *λ*_ex_ = 310 nm. Red bars represent the intensity with subsequent addition of GSH (20 *μ*M).

Besides sensitivity, selectivity is another very important parameter to evaluate the performance of a new fluorescent probe in the presence of amino acids. Under the same conditions, the fluorescence changes of **KL-DN** upon addition of various amino acids and sulfide were almost negligible within 2 min. Only GSH and Cys promoted fluorescent signal enhancement at 487 nm (Figure 3). Interestingly, **KL-DN** showed no fluorescence change when responding to the same amount of sulfide and Hcy. No absorption spectral change of **KL-DN** added sulfide and Hcy was obtained (Figure S11). We tried to test fluorescence spectra of **KL-DN** (10 *μ*M) upon addition of excess Na_2_S (50 eq) and Hcy (20 eq) for 45 min. In fact, Na_2_S and Hcy enabled reacting with azide to induce the generation of amine, but the reaction rate was too slow to interfere with GSH detection (Figures S12–S13). We also studied the NPA charges on S of •OH for Path R4 in the gas (a) and aqueous phases (b) of these three thiols through theoretical calculation. The result was that the attacking ability of Hcy was weakest after losing protons (Table S1). Furthermore, we attempted to detect other possible reducing species existing in biological samples. The test results indicated that Fe^2+^ and ascorbate showed no fluorescence enhancement, which suggested that **KL-DN** displayed excellent selectivity for GSH and Cys detection (Figure S14).

### 2.4. Reaction Mechanism

As far as we know, the probes with azido were found to be very sensitive sensors for sulfide due to the reduction of an azido to amidogen. There were a few literatures reporting that an azido could be reduced by thiols [10]. Because of this, we were interested in the mechanism and devoted to researching the product from the reaction between **KL-DN** and GSH. The results of nuclear magnetic titration indicated that no substitution on the aromatic ring was observed upon the gradual addition of GSH. As shown in Figure S15, the obvious upfield shifts from 8.1 ppm to 7.95 ppm for the aromatic protons near the azido were obtained on account of the reduction of azido. And then, we preformed the chemical reaction of **KL-DN**-GSH and **KL-DN**-Cys with the result of the formation of compound **4** detected by TLC. Next, high-performance liquid chromatography analysis (HPLC) also showed that there was the same peak with the same retention time between **KL-DN**-GSH and compound **4** under the same condition (Figure S16). The result of HPLC displayed that there was no new peak after addition of Hcy (Figure S17), which illustrated that Hcy was unable to lead to reduction of **KL-DN** within a short time. The LC-MS spectrometry was used to confirm the product of **KL-DN**-GSH and **KL-DN**-Cys (Figures S18–S21). The molecular weight of the product with retention time of 15.70 min was the same as the molecular weight of compound **4**, which was in accord with the mechanism of the reported literature and the result of HPLC. Subsequently, we confirmed the feasibility of the reaction through the density functional theory (DFT) calculation. The energy gaps of **KL-DN** between LUMO and HOMO were smaller than compound **4** (Figure S22), revealing that **KL-DN** was capable of transforming into compound **4**.

### 2.5. Imaging of GSH Presence in HeLa Cells

**Figure 4:**
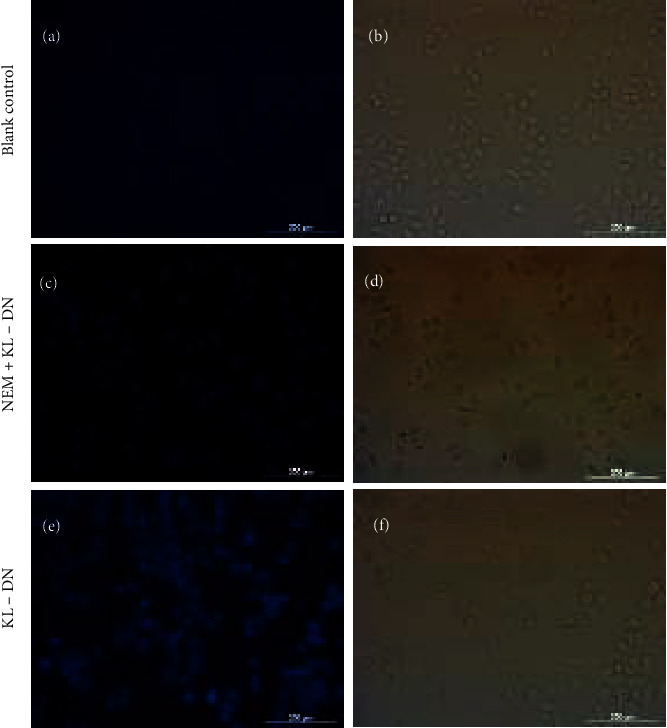
Microscopic images of HeLa cells. (a) Blank control. (b) Corresponding bright field image. (c) Preincubation with 5 mM NEM thiol scavenger for 1 h at 37°C, followed by incubation with 40 *μ*M **KL-DN** for 3 h at 37°C. (d) Corresponding bright field image. (e) Incubation for 3 h at 37°C with 40 *μ*M **KL-DN**. (f) Corresponding bright field image. Scale bar = 200 *μ*m.

In order to show the advantage of **KL-DN**, a research was undertaken to examine the ability of **KL-DN** to act as an imaging agent for endogenous GSH in HeLa cells. As shown in Figure 4, HeLa cells were incubated with 40 *μ*M **KL-DN** for 3 h at 37°C and subsequently washed with PBS (pH 7.4). As the negative-control experiment, HeLa cells were preincubated with 5 mMN-ethylmaleimide (NEM) at 37°C for 1 h to scavenge thiols in the HeLa cells, and then the cells were washed with PBS to remove excess NEM, followed by incubation with 40 *μ*M **KL-DN** for 3 h. In blank-control experiment, no fluorescence was obtained (Figure 4(a)). Exposed to **KL-DN**, HeLa cells exhibited strong fluorescence (Figure 4(e), without NEM), while there was no evident fluorescence in the negative control cells that were pretreated with NEM (Figure 4(c)). Thus, the electrically neutral **KL-DN** was able to enter cells and indeed triggered by intracellular GSH in a selective fashion. Furthermore, **KL-DN** and compound **4** with the skeleton of *β*-carboline were necessary to be studied their cytotoxicity against HeLa cells. After modification by azido, **KL-DN** exhibited low toxicity against HeLa cells after 48 h via MTT method (Figure S23). Interestingly, compound **4** showed negligible toxicity against HeLa cells (Figure S24), which revealed that **KL-DN** and compound **4** had the potential to become imaging agents in cancer cells.

### 2.6. Fluorescence Imaging of GSH in Wheat Germ Slice Tissues

**Figure 5:**
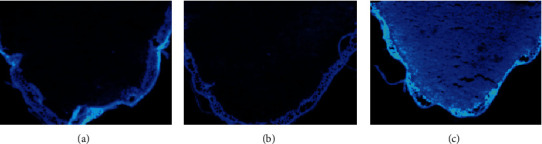
The fluorescent images of wheat germ slice tissues. (a) Blank experiment. (b) The fluorescent images of wheat germ slice tissues pretreated with NEM (0.5 mM) for 5 min and then incubated with **KL-DN** (20 *μ*M) for 5 min. (c) The fluorescent images of wheat germ slice tissues incubated with **KL-DN** (20 *μ*M) for 5 min. Scale bar = 500 *μ*m.

To further investigate the other biological applications, the fluorescence imaging of **KL-DN** was carried out in wheat germ slice tissues where abundant GSH exists in wheat germ (100 mg/g). First of all, the wheat seeds (Xiaoyan 22) were chosen and activated through soaking for 24 h. Then, the sprouted wheat seeds were sliced piece by piece via freezing microtome. The unbroken slices were used for fluorescence imaging. In the control experiment, the wheat germ slice was pretreated with the thiol blocking reagent N-ethylmaleimide (NEM) for 5 min and then incubated with **KL-DN** (20 *μ*M) for 5 min. The fluorescent microscopy image of the slice showed no fluorescence (Figure 5(b)). The conspicuous fluorescence was observed after adding **KL-DN** (20 *μ*M) (Figure 5(c)). These results indicated that **KL-DN** was capable of reacting with endogenous GSH with high sensitivity to produce discernible fluorescence responses in the wheat germ tissues.

## 3. Conclusion

In summary, we have designed and synthesized a new 6-azido *β*-carboline alkaloid derivative whose fluorescent signal was turned on via redox reaction of quenched group (azido) with biological GSH. With a low limit of detection (92 nM), the probe **KL-DN** exhibited high selectivity and rapid response toward GSH and Cys when compared to other amino acids and simple gasotransmitter H_2_S. Furthermore, all these features made **KL-DN** favorable for direct monitoring of GSH in the wheat germ tissues and HeLa cells, demonstrating its practical application in biological systems with high figures of merit. We believe that this new probe will be of great benefit for many researchers engaging in the study of the effect of GSH on plant organisms.

## Data Availability

The data used to support the findings of this study are available from the corresponding author upon request.

## Supplementary Materials

Supplementary Materials
*Figure S1*. Synthetic route. *Figure S2*. The ^1^H NMR spectra of compound **4**. *Figure S3*. The ^13^C NMR spectra of compound **4**. *Figure S4*. The mass spectrum of compound **4**. *Figure S5*. The ^1^H NMR spectra of **KL-DN**. *Figure S6*. The ^13^C NMR spectra of **KL-DN**. *Figure S7*. The mass spectrum of **KL-DN**. *Figure S8*. (A) The fluorescence intensity change of **KL-DN** (10 *μ*M) at 487 nm upon addition of GSH, indicative of good linear relationship. (B) The photo of **KL-DN** in the absence and presence of 2 eq of GSH, which is excited by a handheld UV lamp (365 nm). *Figure S9*. Job's plot for a 1 : 1 stoichiometry between **KL-DN** and GSH in DMSO/PBS buffer (1 : 1, v/v, pH 7.4), *λ*ex = 310 nm. *Figure S10*. The effect of pH from 2 to 12 on the fluorescence signal changes of **KL-DN** (10 *µ*M) at 487 nm in the absence and presence of 2 equivalents of GSH in DMSO/PBS buffer (1 : 1, v/v, pH 7.4), *λ*ex = 310 nm. *Figure S11*. The absorption spectrum change of **KL-DN** (10 *μ*M) upon addition of 2 equivalents of various reducers in DMSO/PBS buffer (1 : 1, v/v, 10 mM, pH 7.4). *Figure S12*. The fluorescence spectral changes of **KL-DN** (10 *μ*M) upon addition of Na_2_S (50 eq), *λ*_ex_ = 310 nm. All the solutions are in DMSO/PBS buffer (1:1, v/v, pH 7.4). *Figure S13*. The fluorescence spectral changes of **KL-DN** (10 *μ*M) upon addition of Hcy (20 eq), *λ*_ex_ = 310 nm. All the solutions are in DMSO/PBS buffer (1 : 1, v/v, pH 7.4). *Figure S14*. The fluorescence intensity change of **KL-DN** (10 *μ*M) at 487 nm upon addition of 2 equivalents of various reducers in DMSO/PBS buffer (1 : 1, v/v, 10 mM, pH 7.4), *λ*ex = 310 nm. *Figure S15*. The changes of chemical shift for the aromatic protons of **KL-DN** upon gradual addition of GSH. *Figure S16*. HPLC analysis of the **KL-DN**-GSH, **KL-DN**-Cys, and compound **4** under the same condition. All the solutions were prefiltered with methanol before HPLC detection. The peaks with retention time of 5.6 and 7.5 min are assigned to the corresponding fluorescent product, respectively. The retention time differed by 0.2 minutes, which belonged to instrument error. *Figure S17*. HPLC analysis of the **KL-DN**, **KL-DN**-Hcy, and compound **4** under the same condition. *Figure S18*. LC-MS analysis of the **KL-DN**-Cys; the solution was preextracted with methanol before LC-MS assay. *Figure S19*. LC-MS analysis of the **KL-DN**-Cys with the retention time; the MS spectrum of peak with retention time of 15.76 min, which is assigned to the corresponding fluorescent product. *Figure S20*. LC-MS analysis of the **KL-DN**-GSH; the solution was preextracted with methanol before LC-MS assay. *Figure S21*. LC-MS analysis of the **KL-DN**-GSH; the MS spectrum of peak with retention time of 15.76 min, which is assigned to the corresponding fluorescent product. *Figure S22*. Electron orbital energy calculation of **KL-DN** and compound **4**. *Figure S23*. Cell viability assay of **KL-DN** incubated in HeLa cell lines for 48 h, and the cell viability was observed via MTT assay. *Figure S24*. Cell viability assay of compound **4** in HeLa cell lines for 48 h, and the cell viability was observed via MTT assay. *Table S1*. The NPA charges on S of •OH for Path R4 in the gas (a) and aqueous phases (b).
