# Supplementary material for: Efficient β-Carboline Alkaloid-Based Probe for Highly Sensitive Imaging of Endogenous Glutathione in Wheat Germ Tissues
Source: Int J Anal Chem. 2020 Sep 15;2020:8675784. doi: 10.1155/2020/8675784 (PMC7512064; doi:10.1155/2020/8675784)
Supplement: Supplementary Materials — Figure S1. Synthetic route. Figure S2. The 1H NMR spectra of compound 4. Figure S3. The 13C NMR spectra of compound 4. Figure S4. The mass spectrum of compound 4. Figure S5. The 1H NMR spectra of KL-DN. Figure S6. The 13C NMR spectra of KL-DN. Figure S7. The mass spectrum of KL-DN. Figure S8. (A) The fluorescence intensity change of KL-DN (10 μM) at 487 nm upon addition of GSH, indicative of good linear relationship. (B) The photo of KL-DN in the absence and presence of 2 eq of GSH, which is excited by a handheld UV lamp (365 nm). Figure S9. Job's plot for a 1 : 1 stoichiometry between KL-DN and GSH in DMSO/PBS buffer (1 : 1, v/v, pH 7.4), λex = 310 nm. Figure S10. The effect of pH from 2 to 12 on the fluorescence signal changes of KL-DN (10 µM) at 487 nm in the absence and presence of 2 equivalents of GSH in DMSO/PBS buffer (1 : 1, v/v, pH 7.4), λex = 310 nm. Figure S11. The absorption spectrum change of KL-DN (10 μM) upon addition of 2 equivalents of various reducers in DMSO/PBS buffer (1 : 1, v/v, 10 mM, pH 7.4). Figure S12. The fluorescence spectral changes of KL-DN (10 μM) upon addition of Na2S (50 eq), λex = 310 nm. All the solutions are in DMSO/PBS buffer (1:1, v/v, pH 7.4). Figure S13. The fluorescence spectral changes of KL-DN (10 μM) upon addition of Hcy (20 eq), λex = 310 nm. All the solutions are in DMSO/PBS buffer (1 : 1, v/v, pH 7.4). Figure S14. The fluorescence intensity change of KL-DN (10 μM) at 487 nm upon addition of 2 equivalents of various reducers in DMSO/PBS buffer (1 : 1, v/v, 10 mM, pH 7.4), λex = 310 nm. Figure S15. The changes of chemical shift for the aromatic protons of KL-DN upon gradual addition of GSH. Figure S16. HPLC analysis of the KL-DN-GSH, KL-DN-Cys, and compound 4 under the same condition. All the solutions were prefiltered with methanol before HPLC detection. The peaks with retention time of 5.6 and 7.5 min are assigned to the corresponding fluorescent product, respectively. The retention time differed by 0.2 minutes, which b [file 8675784.f1.doc]

| **Efficient β-carboline alkaloid-based probe for highly sensitive imaging of endogenous glutathione in wheat germ tissues**  Xiaohui Ji^a^, Dan Zhang^a^, Le Li^b*^, Lingxia Jin^a^, Rui Wu^a^  *a. Shaanxi Province Key Laboratory of Catalytic Foundation and Application, School of Chemistry and Environment Science, Shaanxi University of Technology, Hanzhong 723001, China, E-mail: zhangdan@snut.edu.cn.*  *b. Shaanxi Key Laboratory of Industrial Automation, School of Mechanical Engineering, Shaanxi University of Technology, Hanzhong 723001, China.* |
| --- |

**Experimental**

**Materials and equipment**

Tryptophan, n-ethyl maleimide, sodium azide and selenium dioxide were obtained from Aladdin Company. Glutathione (GSH), cysteine (Cys), homocysteine (Hcy), glycine (Gly), tyrosine (Tyr), serine (Ser), lysine (Lys), threonine (Thr), alanine (Ala), citrulline, methionine (Met), glutamic (Glu), cysteine, proline (Pro), leucine and histidine (His) were purchased from Voerson Reagent Company and used without further purification. All solvents were of reagent grade. Water used was ultrapure water.

Absorption and emission spectra were measured by the Shimadzu 1750 UV visible spectrometer and the RF-5301 fluorescence spectrometer (Japan), respectively. LC-MS spectra was connected by using API 2000 high performance liquid chromatography-mass spectrometer (UK). NMR spectra were collected on a Bruker 500 avance III spectrometer. Mass spectrometric (MS) data were obtained with ESI-MS instruments. The thin-layer chromatography (TLC) was carried out on silica gel plates (60 F-254) using UV-light to monitor the reaction (254 nm and 365 nm). Fluorescence images of wheat germ slice and live cells were performed by fluorescence microscopy (LEICA DM6 B).

**Synthetic section**

Preparation of compound **1**

Tryptophan (2 g) was added into ultrapure water (80 mL). Then 0.01 M H_2_SO_4_ was added drop by drop until the tryptophan was dissolved completely. We mixed 37% formaldehyde aqueous solution (3.2 mL) into the above system and stirred at room temperature for 6 h. After white precipitate separated out, the pH was changed from 4 to 6. Mixed solution was placed at 4°C overnight. After filtered, the white precipitate was washed by ultrapure water (10 mL) for 3 times to get compound **1** (white solid, 1.78 g, 85%).

Preparation of compound **2**

The compound **1** (1 g) with SeO_2_ (500 mg) was stirred in 100 mL acetic acid at 150°C for 24 h. Then the cooled mixture was evaporated under reduced pressure. After we added 60 mL ultrapure water, the pH was changed from 3 to 9. We extracted the product using ethyl acetate (40 mL, 3 times) and separated the crude product by silica gel column chromatography to get compound **2** (yellowish-white solid, 303 mg, 39%).

Preparation of compound **3**

The compound **2** (300 mg) was dissolved in 4 mL CF_3_COOH, then NaNO_3_ (700 mg) was added and stirred at room temperature for 18 h. We added 30 mL ultrapure water to separate out the yellow precipitate. As a result, the pH was changed from 2 to 9. After the filtered of aqueous solution, the yellow precipitate was washed by ultrapure water (10 mL, 3 times) to get the crude product. The crude product was then separated by silica gel column chromatography with the eluent (ethyl acetate: petroleum ether=1:1). Consequently, the compound **3** was obtained with the yield 50% (yellow solid, 190 mg, 50%).

Preparation of compound **4**

The compound **3** (300 mg) was dissolved in 40 mL methyl alcohol, then hydrazine hydrate (100 mg) and Pd/C (catalyst, 50 mg) were added and stirred at 90°C for 6 h. After the filtering of Pd/C, the cooled mixture was evaporated under reduced pressure. The crude product was separated by silica gel column chromatography with the eluent (DCM:methyl alcohol=15:1) to obtain compound **4** with the yield of 85% (Yellow solid, 219 mg, 85%).

Preparation of **KL-DN**

The pure compound **4** (100 mg) was dissolved in 1 mL DMF in ice-salt-bath. Then 1 mL 30% HCl was added into the above solution drop-by-drop. After 3 min, NaNO_2_ (113 mg) dissolved in 5 mL distilled water was added drop-by-drop at 0°C. After 40 min, we mixed NaN_3_ (106 mg) dissolved in 5 mL distilled water into the above-mentioned mixture. The mixture solution was stirred at room temperature overnight. After pH was changed from 2 to 8, the precipitate was filtered, washed by distilled water. The obtained crude product was separated by silica gel column chromatography to get **KL-DN** (Fig. S2) (yellow solid, 60 mg, 53%). ^1^H NMR (500 MHz, DMSO-*d*) δ 11.70 (s, 1H), 8.91 (s, 1H), 8.34 (d, J=5.2 Hz, 1H), 8.15 (d, J=12.8 Hz, 1H), 8.08 (d, J=1.9 Hz, 1H), 7.63 (d, J=8.7 Hz, 1H), 7.29 (dd, J=8.7, 2.2 Hz, 1H); ^13^C NMR (125 MHz, DMSO-*d*) δ 138.61, 137.1, 134.9, 131.4, 127.3, 122.1, 120.5, 115.6, 113.9, 112.3.

**Sample preparation and titration**

The concentration of stock various amino acids and sodium sulfide were 5 mM in deionized water. Stock solution of **KL-DN** (1 mM) were prepared in DMSO and then further diluted to 10 μM for titration experiments. An appropriate volume of each analyte was added to the test solution every time. UV and fluorescent spectra were measured within 2 min.

**Cell incubation and imaging**

HeLa cells were treated in DMEM supplemented with FBS (10% fetal bovine serum) in an atmosphere of 5% CO_2_ at 37°C. The cells were plated on glass sheet and allowed to adhere for 12 h. First, the HeLa cells were incubated with 40 μM **KL-DN** for 3 h at 37°C after washed with PBS buffer solution for three times. Second, the HeLa cells were pretreated with 5 mM NEM at 37°C for 1 h, washed with PBS for three times, then incubated with 40 μM **KL-DN** at 37°C for 3 h. Finally, the cells were analyzed by fluorescent microscope.

Calculation of fluorescence quantum yield

By applying the equation, the fluorescence quantum yield of the sample was calculated using quinine sulfate as the standard (Φ=0.54, 0.1 M H_2_SO_4_). In this equation, Φ_unk_ and Φ_std_ represent the fluorescence quantum yield of the sample and the standard, respectively. I_unk_ and I_std_ are the integral area of fluorescent spectra, respectively. A_unk_ and A_std_ are the absorbance of the sample and the standard at the excitation wavelength. n_unk_ and n_std_ are the refractive indexes of the corresponding solution.

Fig. S1 Synthetic route

Fig. S2 The ^1^H NMR spectra of compound **4**

Fig. S3 The ^13^C NMR spectra of compound **4**

Fig. S4 The mass spectrum of compound **4**

Fig. S5 The ^1^H NMR spectra of **KL-DN**

Fig. S6 The ^13^C NMR spectra of **KL-DN**

Fig. S7 The mass spectrum of **KL-DN**

B


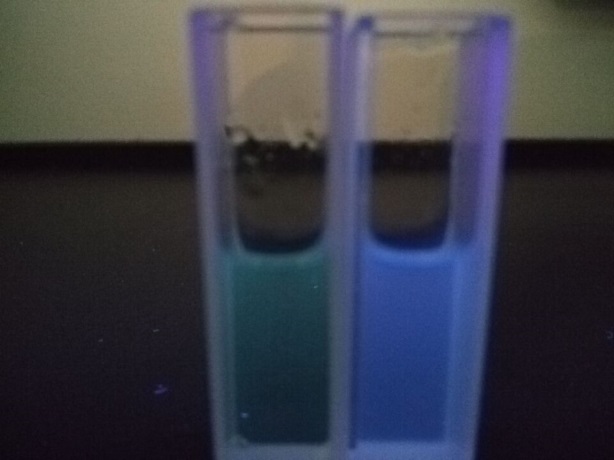


A

B

Fig. S8 (A) The fluorescence intensity change of **KL-DN** (10 μM) at 487 nm upon addition of GSH, indicative of good linear relationship. (B) The photo of **KL-DN** in the absence and presence of 2 eq of GSH, which is excited by a hand-held UV lamp (365 nm).

Fig. S9 Jobs plot for a 1:1 stoichiometry between **KL-DN** and GSH in DMSO/PBS buf fer (1:1, v/v, pH 7.4), λex=310 nm.

Fig. S10 The effect of pH from 2 to 12 on the fluorescence signal changes of **KL-DN** (10 µM) at 487 nm in the absence and presence of 2 equivalents of GSH in DMSO/PBS buffer (1:1, v/v, pH 7.4), λex=310 nm.

Fig. S11 The absorption spectrum change of **KL-DN** (10 μM) upon addition of 2 equivalents of various reducers in DMSO/PBS buffer (1:1, v/v, 10 mM, pH 7.4).

Fig. S12 The fluorescence spectral changes of **KL-DN** (10 μM) upon addition of Na_2_S (50 eq), λ_ex_=310 nm. All the solutions are in DMSO/PBS buffer (1:1, v/v, pH 7.4).

Fig. S13 The fluorescence spectral changes of **KL-DN** (10 μM) upon addition of Hcy (20 eq), λ_ex_=310 nm. All the solutions are in DMSO/PBS buffer (1:1, v/v, pH 7.4).

Fig. S14 The fluorescence intensity change of **KL-DN** (10 μM) at 487 nm upon addition of 2 equivalents of various reducers in DMSO/PBS buffer (1:1, v/v, 10 mM, pH 7.4), λex = 310 nm.

Fig. S15 The changes of chemical shift for the aromatic protons of **KL-DN** upon gradual addition of GSH.

Fig. S16 HPLC analysis of the **KL-DN**-GSH, **KL-DN**-Cys and Compound **4** under the same condition. All the solutions were pre-filtered with methanol before HPLC detection. The peaks with retention time of 5.6, 7.5 min are assigned to the corresponding fluorescent product, respectively. The retention time differed by 0.2 minutes, which belonged to instrument error.

Fig. S17 HPLC analysis of the **KL-DN**, **KL-DN**-Hcy and compound **4** under the same condition.

Fig. S18 LC-MS analysis of the **KL-DN**-Cys; the solution was pre-extracted with methanol before LC-MS assay.

Fig. S19 LC-MS analysis of the **KL-DN**-Cys with the retention time; the MS spectrum of peak with retention time of 15.76 min, which is assigned to the corresponding fluorescent product.

Fig. S20 LC-MS analysis of the **KL-DN**-GSH; the solution was pre-extracted with methanol before LC-MS assay.

Fig. S21 LC-MS analysis of the **KL-DN**-GSH; the MS spectrum of peak with retention time of 15.76 min, which is assigned to the corresponding fluorescent product.

**KL-DN**  **Compound 4**


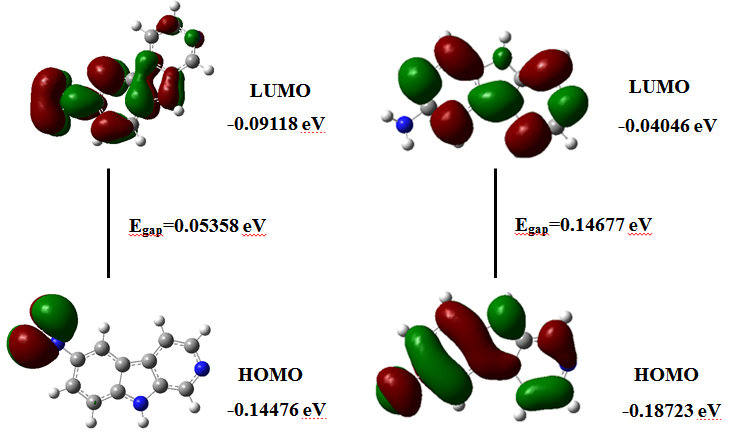


Fig. S22 Electron orbital energy calculation of **KL-DN** and compound **4**

Fig. S23 Cell viability assay of **KL-DN** incubated in HeLa cell lines for 48 h, and the cell viability was observed via MTT assay.

Fig. S24 Cell viability assay of compound **4** in HeLa cell lines for 48 h, and the cell viability was observed via MTT assay.

|  | Cys | Hcy | GSH |
| --- | --- | --- | --- |
| ρ_S_ | -0.05392 | -0.05960 | -0.05320 |

Table S1 The NPA Charges on S of •OH for Path R4 in the Gas (a) and Aqueous Phases (b).
